# Insulin Pathway Changes in Localized Prostate Cancer: A Multi-Institutional Analysis

**DOI:** 10.3390/cancers18101636

**Published:** 2026-05-19

**Authors:** Evan R. Adler, Anwaruddin Mohammad, Pankaj Kumar, Robert J. Rounbehler, Michelle L. Churchman, Laura S. Graham, Eric A. Singer, Bodour Salhia, Adanma Ayanambakkam, Kenneth G. Nepple, Zin W. Myint, Qiang Li, Saum Ghodoussipour, Jennifer M. King, G. Daniel Grass, Sumati V. Gupta, Paul V. Viscuse

**Affiliations:** 1 Department of Internal Medicine, University of Virginia, Charlottesville, VA 22904, USA; nfm6jm@uvahealth.org; 2 Department of Public Health Sciences, University of Virginia, Charlottesville, VA 22904, USA; 3 Aster Insights, Hudson, FL 34667, USA; 4 Department of Internal Medicine, University of Colorado Cancer Center, Aurora, CO 80045, USA; 5 Department of Urology, The Ohio State University Comprehensive Cancer Center, Columbus, OH 43210, USA; 6 Department of Cancer Biology, Norris Comprehensive Cancer Center, University of Southern California, Los Angeles, CA 90033, USA; 7 Department of Internal Medicine, Stephenson Cancer Center, The University of Oklahoma Health Sciences, Oklahoma City, OK 73104, USA; 8 Department of Urology, University of Iowa and Clinic Holden Cancer Center, Iowa City, IA 52242, USA; 9 Division of Medical Oncology, Department of Internal Medicine, University of Kentucky, Lexington, KY 40506, USA; 10 Department of Urology, Roswell Park Comprehensive Cancer Center, Buffalo, NY 14203, USA; 11 Department of Surgery, Rutgers Cancer Institute of New Jersey, New Brunswick, NJ 08903, USA; 12 Department of Internal Medicine, Indiana University Melvin and Bren Simon Comprehensive Cancer Center, Indianapolis, IN 46202, USA; 13 Department of Radiation Oncology, H. Lee Moffitt Cancer Center and Research Institute, Tampa, FL 33612, USA; 14 Department of Medicine, Huntsman Cancer Institute, University of Utah, Salt Lake City, UT 84112, USA

**Keywords:** insulin signaling, insulin resistance, AKT-mTOR-PI3K signaling, PTEN, prostate cancer, localized, RNA expression, transcriptome

## Abstract

Background: Prostate cancer is a heterogeneous disease with variable clinical outcomes. If localized, the patient may be cured. However, prostate cancer is lethal if recurrence/progression to metastatic castrate resistant disease occurs. Thus, there is an unmet need to further understand the molecular underpinnings of this progression. Epidemiologic studies show that increased risk of developing and dying from prostate cancer has been associated with elevated serum IGF-1 levels, hyperinsulinemia and metabolic syndrome. Alterations in insulin pathway genes, such as *PTEN*, *FOXO*, and *PIK3CA*, are mutated in up to 32%, 15%, and 11% of localized prostate tumors, respectively. We aimed to further characterize expression of insulin pathway genes in localized prostate cancers in an effort to (1) provide insights into potential mechanisms of progression to metastatic disease and (2) try to further enrich for those prostate tumors that portend worse survival outcomes. Methods: Using the multi-institutional Oncology Research Information Exchange Network (ORIEN) database, gene expression data was analyzed from localized prostate cancer tumors. The raw counts were first normalized, and 176 genes related to the insulin receptor and its downstream pathways were then subset and used for clustering using the non-negative matrix factorization (NMF). The NMF cluster analysis was performed in an attempt to separate gene expression into two groups. Gene Set Enrichment Analysis (GSEA) was then performed between the two groups that had been separated by cluster analysis to determine homology between other GSEA sets. Kaplan–Meier curves were used to assess median overall survival. Cox analysis was performed to generate the adjusted KM curve. Mediation analysis was conducted to determine the relationship between cluster status, TN stage, and survival. Results: Cluster analysis revealed two distinct groups of insulin gene expression, cluster 1 (*n* = 96) and cluster 2 (*n* = 337). Compared with cluster 2, cluster 1 consisted of decreased expression of *PTEN* (*p* < 0.001) and *PIK3R1* (*p* < 0.001), along with increases in the expression of *AKT1* (*p* < 0.001), *IRS1/2* (*p* < 0.001), *FASN* (*p* < 0.001), *IGFBP2* (*p* < 0.001), and *MTOR* (*p* < 0.001). GSEA analysis revealed changes in lipid metabolism and WNT secretion pathways in cluster 1. Cluster 2 GSEA showed pathway changes related to DNA damage repair and testosterone. Patient characteristics between clusters differed significantly in the T and N stages of tumor but not in other ways. In unadjusted analysis, median overall survival was estimated at 117 months and 232 months for cluster 1 and cluster 2, respectively (*p* < 0.05). The proportion of patients who went on to develop metastases (*p* < 0.05) or need chemotherapy (*p* < 0.05) was increased in cluster 1 compared to cluster 2. Repeat survival analysis adjusted for confounders (T stage, N stage, age at diagnosis, pathologic grade) showed no difference in survival between clusters. Mediation analysis showed that the contribution of cluster status to survival was independent of T or N stage. Conclusions: A subset of localized prostate cancer patients demonstrated linked insulin pathway changes that are consistent with prior studies describing a pattern of insulin dysregulation. Though the group characterized by insulin dysregulation initially showed worse survival outcomes, this difference disappeared when controlling for confounders. Though baseline differences in tumor stage seemed to most readily explain the difference in survival between clusters, mediation analysis showed that the effect of cluster status on survival was independent of tumor stage. This suggests that other confounders, such as pathologic grade or baseline age, may explain the survival difference.

## Simple Summary

Dysfunction of insulin pathway genes is known to occur in prostate cancers. There is a lack of large studies characterizing exactly what transcriptome changes are involved and their impact on clinical outcomes. Focusing on localized cancers specifically, we used a multi-institutional database to look for differences in RNA expression for these insulin pathway genes. We were able to identify two RNA expression groups—one with changes consistent with pathway dysfunction and the other not. Though on an initial unadjusted analysis, the dysfunction group showed significantly reduced overall survival, this disappeared when controlling for confounders. These findings imply that confounding factors likely explain the difference in survival.

## 1. Introduction

Prostate cancer is a heterogeneous disease with variable clinical outcomes [1]. If localized, the patient may be cured [1,2]. However, prostate cancer is typically incurable once metastatic disease develops. Despite variable responses to androgen receptor (AR) targeted therapy for castrate sensitive prostate cancer, the disease is ultimately lethal following progression to castrate resistance [1,2]. Thus, there is an unmet need to further understand the molecular underpinnings of this progression.

Insulin pathway changes have been observed in a variety of cancers [3,4]. In particular, the PI3K-AKT-mTOR pathway is frequently dysregulated. This key pathway is located downstream of insulin and plays a crucial role in cell growth, metabolism, drug resistance, and metastasis [5]. It has a prognostic role in renal, colorectal, pancreatic, endometrial, breast, and brain cancers [6]. As a result of the commonality of this molecular dysfunction across malignancies, a number of FDA-approved drugs involving this pathway have been developed, including the PI3K-, AKT-, and mTOR-inhibitors alpelisib, capivasertib, and everolimus, respectively [7,8,9]. These are used to treat cancers as varied as breast cancers and lymphomas [7,8,9].

With respect to prostate cancer, epidemiologic studies have shown an increased risk of developing prostate cancer with both hyperinsulinemia and elevated serum IGF-1 levels [10,11,12]. Hyperinsulinemia is particularly relevant to prostate cancer, as androgen deprivation therapy (ADT) is the first-line treatment for most prostate cancers and has been shown to rapidly induce hyperinsulinemia via insulin resistance after about 2 weeks of treatment [13,14]. Once present, hyperinsulinemia is associated with shorter time to the development of castrate-resistant disease and increased cancer-related mortality [15,16].

Insulin pathway changes are common in prostate cancer. PTEN, an important regulator of PI3K, is altered in up to 60% of localized prostate tumors [17]. PIK3R1, a subunit of the PI3K complex, undergoes genomic loss in 24% of localized prostate tumors and 36% of metastatic tumors [18]. On a broader level, the PI3K-Akt-mTOR pathway is upregulated in 30–50% of prostate cancers [19,20]. This pathway is located downstream of the insulin receptor and terminates with mTOR, an oncogene that promotes cell growth [21]. These molecular changes in insulin pathways have implications for progression to metastatic disease and therapy resistance [17]. Homozygous *PTEN* loss is associated with both metastatic progression and androgen insensitivity [22,23,24]. PI3K-Akt-mTOR signaling promotes angiogenesis, motility, and epithelial-to-mesenchymal transition (EMT)—all key processes in the progression from localized to metastatic disease [25]. Insulin itself has been found to promote migration and invasiveness in prostate cancer cells via the transcription factor FOXC2, which is downstream of both PI3K and ERK1/2 [26]. Importantly, there exists a reciprocal relationship between AR signaling and PI3K-Akt-mTOR in that AR inhibition is associated with upregulation of PI3K-Akt-mTOR and vice versa [27,28]. This is particularly relevant given the ubiquity of AR inhibition as first-line treatment for many prostate cancers [27,28].

Emerging clinical trial data indicate that targeting PI3K-AKT signaling may be beneficial in *PTEN*-deficient prostate cancer [29,30]. In the phase 3 IPATential150 study, treatment with the *PI3K* inhibitor ipatasertib plus abiraterone resulted in a statistically significant improvement in radiographic progression-free survival (rPFS) compared with placebo plus abiraterone among the subset of patients with *PTEN*-deficient metastatic castrate resistant prostate cancer (mCRPC); however, this did not translate into an overall survival benefit and was associated with higher rates of treatment-related toxicity [29]. Similarly, the phase 3 CAPItello-281 trial demonstrated improved rPFS with the *AKT*-inhibitor capivasertib plus abiraterone compared to placebo and abiraterone in *PTEN*-deficient metastatic hormone-sensitive prostate cancer (mHSPC), although overall survival data remain immature, and increased adverse events were observed with combination therapy [30].

The existing data is suggestive of a role of PI3K-Akt-mTOR signaling in disease progression, particularly with respect to the development of metastases and androgen resistance [22,23,24,25,26]. Therapeutic targeting of this pathway has demonstrated benefit across other cancers, and there are early but positive results in prostate cancer [7,8,9,29,30]. Still, there exists a need for large studies connecting insulin pathway transcriptome changes, including those associated with PI3K-Akt-mTOR signaling, with clinical outcomes. By leveraging a large multi-institutional database, we aimed to characterize insulin pathway RNA changes present in localized prostate cancers. By focusing on localized disease, the goal was to identify early transcriptomic changes that might predispose patients to developing more advanced disease. We aimed to focus on the known dysregulation in PI3K-Akt-mTOR signaling, but also chose to include a broader cassette of genes downstream of the insulin receptor or related to regulation of its function. This was done in an attempt to capture new associations and enhance our understanding of the molecular dysfunction that occurs at the gene expression level. The specific aims of the study were to (1) provide insights into the molecular dysfunction occurring at the transcription level of insulin pathway genes and (2) assess for associations between insulin pathway transcriptome dysregulation and clinical outcomes.

## 2. Materials and Methods

### 2.1. Data Source

We used a multi-institutional database known as Oncology Research Information Exchange Network (ORIEN) to access clinical and molecular information about patients with localized prostate cancer. The database was established in 2014 and represents a collaboration between 18 cancer centers across the United States. Through the ORIEN database, patient data is collected under a Total Cancer Care (TCC) protocol. With this TCC protocol, research participants consent to having their de-identified data included in a searchable database for providers that includes clinical characteristics, germline DNA, and tumor DNA information. In addition to molecular data, the ORIEN database contains clinical and demographic information about participants. For this study, the following clinical and demographic parameters were analyzed: age, race, gender, body mass index, co-morbid conditions, cancer diagnosis, histology, tumor stage, tumor grade, and medications. The information contained in the database is inferred from patient questionnaires as well as patient samples, which may include tumor, blood, and/or other specimens. As a study approved by the ORIEN GURIG (Genitourinary Research Interest Group), this research was included under the previously approved TCC (Total Cancer Care) protocol.

### 2.2. Patient Cohort

In the ORIEN database, patients were first identified as having both localized prostate cancers as well as the requested RNA sequencing data and/or whole exome sequencing (WES) data (*n* = 1495). Patients with metastatic disease were excluded, leaving *n* = 456 patients with M0 disease available to be considered in the analysis.

### 2.3. RNA Sequencing and Non-Negative Matrix Factorization

We chose 176 insulin pathway genes for analysis. These were accumulated from established GSEA sets, including the following: “REACTOME INSULIN RECEPTOR SIGNALING CASCADE,” “REACTOME SIGNALING BY INSULIN RECEPTOR,” “KEGG INSULIN SIGNALING PATHWAY,” and “HP INSULIN RESISTANCE.” The following additional genes were not in the aforementioned GSEA sets but were included because of their relevance in insulin signaling based on the reference publication: *PTEN*, *IGFBP1*, *IGFBP3*, *IGF-1R*, *MDM2*, *CASP9*, *CDKN1A*, *CDKN1B* [31]. The raw counts were first normalized, and 176 genes related to the insulin receptor and its downstream pathways were then subsetted and used for clustering using non-negative matrix factorization (NMF) [32]. NMF cluster analysis was performed in an attempt to separate gene expression into two groups (k = 2). Two groups, rather than a greater number, were chosen in an attempt to capture generalized differences in pathway expression. Supplementary Figure S1 demonstrates a comparison of silhouette plots for k = 2 compared to k = 3 to further justify the choice of k = 2. The silhouette plot for k = 2 shows a greater number of positive values, indicating better quality clustering with a narrower distribution of values.

Following this step, a two-dimensional scatter plot (Figure S2) was generated to visualize cluster separation. This showed a subset of samples that demonstrated intermediate membership in both cluster 1 and cluster 2. Specifically, samples with cluster 1 coefficients between 0.0030 and 0.0035 were in this overlapping zone. These values, amounting to 23 patients, were identified as outliers and filtered from the data set. This filtering was performed to improve cluster purity and ensure more robust downstream comparisons by excluding samples with ambiguous membership. The remaining 433 patients were subsequently used as the input for differential gene expression analysis between the two groups using the DESeq2 package. *p*-values used to delineate differences in gene expression were adjusted to an FDR (False Discovery Rate) of 0.05.

### 2.4. Gene Set Enrichment Analysis

For the Gene Set Enrichment Analysis (GSEA), Molecular Signatures Database hallmark, C2, and C5 gene sets were considered as references. The QuSAGE R package version 3.23 [33] was used to conduct the GSEA analysis. *p*-values used to delineate differences in gene pathway expression were adjusted to an FDR of 0.25. A more lenient FDR was chosen for this step in order to capture broader changes in pathway expression for this initial exploratory analysis.

### 2.5. Clinical Analyses

Kaplan–Meier curves were used to assess any survival differences between the two groups of patients. For the descriptive table, *p*-values were calculated using the Kruskal–Wallis test for Age and BMI and Fisher’s exact test for all other variables. Fisher’s exact test was also utilized for comparisons between the percentages of patients who developed metastases or used chemotherapy between the two groups. *p*-values used in the descriptive table and for Fisher’s exact test were unadjusted. The Cox model was used to assess the impact of co-variates on survival. The co-variates included were patient age at diagnosis, tumor T stage, tumor N stage, pathological tumor grade, and cluster status. A KM curve between clusters was generated, controlling for the other co-variates: tumor T stage, tumor N stage, age, and pathologic grade. A nomogram demonstrating the relative impact of each co-variate was also included.

### 2.6. Mediation Analysis

Mediation analysis was conducted that included mediator model, outcome model, and decomposing effects. The mediator model utilized logistic regression, and the outcome model utilized Cox proportional hazards. The analysis focused on the relationship between T and N stage with respect to cluster status and overall survival. *p*-values used in this analysis were unadjusted.

## 3. Results

**Figure 1:**
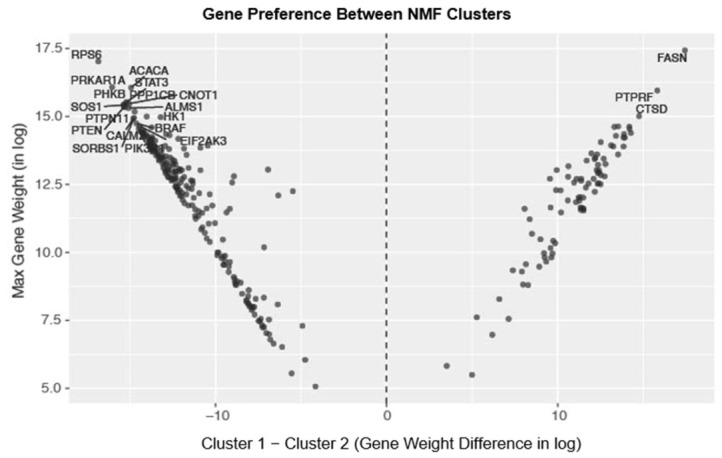
Gene preference plot that displays NMF gene-weight distribution between the two clusters. The x-axis represents the difference in gene weight (on a log scale) between cluster 2 and cluster 1. The y-axis represents the maximum gene weight (on a log scale) for each gene. Genes on the far left (e.g., *RPS6*, *PRKAR1A*) are highly expressed in cluster 2, while genes on the far right (e.g., *FASN*, *PTPRF*, *CTSD*) are highly expressed in cluster 1.

**Figure 2:**
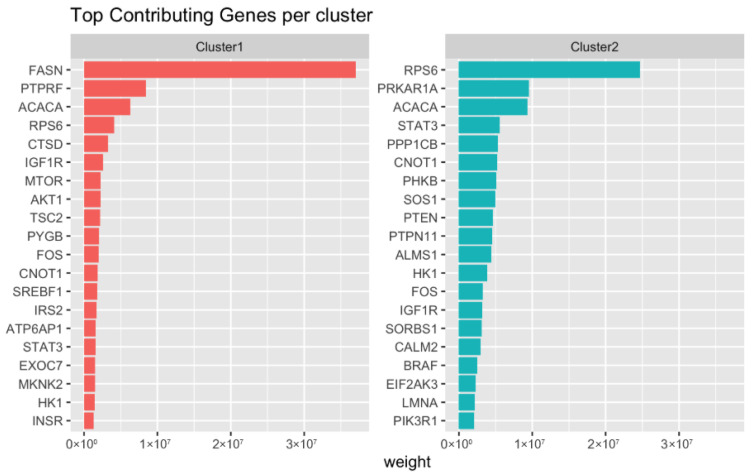
Bar chart comparing the top contributing genes for each of the two different clusters derived from the NMF clustering and gene expression analysis. The chart shows the relative weight attributable to various genes within each cluster. The top 20 genes in each cluster are shown. Notable high contribution genes in cluster 1 include *FASN*, *ACACA*, *IGFR1*, *MTOR*, and *AKT1*. Similarly, notable high contribution genes in cluster 2 include *PTEN*, *ACACA*, *PIK3R1*, and *IGFR1*.

Cluster analysis performed on the total set of localized prostate tumors (*n* = 456) revealed two distinct groups of insulin gene expression, cluster 1 (*n* = 96) and cluster 2 (*n* = 337). The top genes contributing to each cluster are shown in Figure 1, along with the distribution of genes between the two clusters. Figure 2 shows the top 20 genes contributing to each cluster.

**Figure 3:**
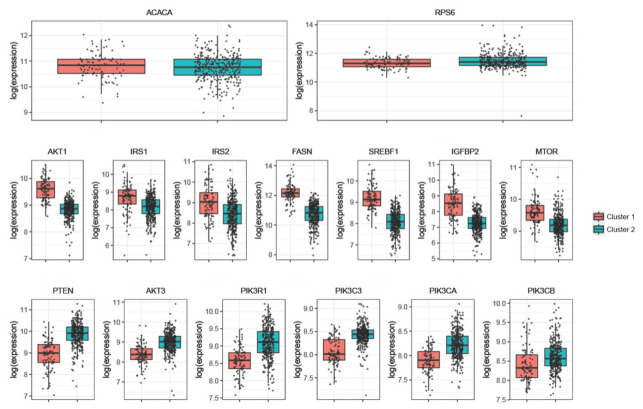
Series of box plots illustrating the normalized expression levels of top-weighted genes across cluster 1 (red) and cluster 2 (teal) for multiple specific genes. The expression is listed on a log scale, and the *p*-value is listed over each box plot. The box plots demonstrate increased expression of *AKT1* (*p* < 0.001), *IRS1*/2 (*p* < 0.001), *FASN* (*p* < 0.001), *SREBF1* (*p* < 0.001), *IGFBP2* (*p* < 0.001), and *MTOR* (*p* < 0.001) in cluster 1 relative to cluster 2. Conversely, *PTEN* (*p* < 0.001), *AKT3* (*p* < 0.001), *PIK3R1* (*p* < 0.001), *PIK3C3* (*p* < 0.001), *PIK3CA* (*p* < 0.001), and *PIK3CB* (*p* < 0.001) are decreased in cluster 1 compared to cluster 2. No difference is noted between *ACACA* or *RPS6* between the two clusters (*p* > 0.05).

Some genes stand out as highly expressed within both clusters. *ACACA* and *RPS6*, for example, are part of the top five contributors to both clusters 1 and 2. However, many other genes demonstrate differential expression between the two groups. Genes significantly elevated in cluster 1 include: *AKT1* (*p* < 0.001), *IRS1/2* (*p* < 0.001), *FASN* (*p* < 0.001), *SREBF1* (*p* < 0.001), *IGFBP2* (*p* < 0.001), and *MTOR* (*p* < 0.001). Those significantly decreased in cluster 1 relative to cluster 2 include: *PTEN* (*p* < 0.001), *AKT3* (*p* < 0.001), *PIK3R1* (*p* < 0.001), *PIK3C3* (*p* < 0.001), *PIK3CA* (*p* < 0.001), and *PIK3CB* (*p* < 0.001) (Figure 3).

**Figure 4:**
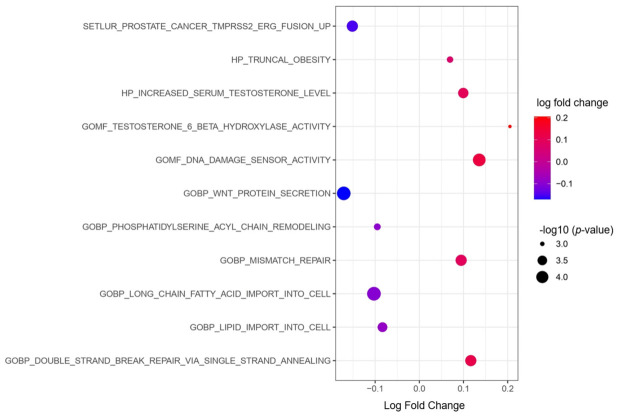
Bubble plot showing the distribution of select pathways across the two clusters. The pathway name is listed on the y-axis, with log change in expression listed on the x-axis. Negative log fold change favors expression in cluster 1, while positive favors cluster 2. All pathways listed demonstrated significant differences in expression (*p*-adjusted < 0.25) and are derived from the C2 and C5 references.

GSEA analysis used the hallmark, C2, and C5 reference data. From these, C5 demonstrated 79 significant pathway changes, C2 had 10, and hallmark had none. The full set of these pathway changes can be found in Tables S1 and S2 for the C5 and C2 datasets, respectively. Select pathway changes that were enriched in cluster 1 include: GOBP_WNT_PROTEIN_SECRETION (*p* = 0.13), GOBP_LONG_CHAIN_FATTY_ACID_IMPORT_INTO_CELL (*p* = 0.13), GOBP_LIPID_IMPORT_INTO_CELL (*p* = 0.16), GOBP_PHOSPHATIDYLSERINE_ACYL_CHAIN_REMODELING (*p* = 0.24), SETLUR_PROSTATE_CANCER_TMPRSS2_ERG_FUSION_UP (*p* = 0.19). Pathways that were enriched in cluster 2 include: GOMF_DNA_DAMAGE_SENSOR_ACTIVITY (*p* = 0.13), GOBP_MISMATCH_REPAIR (*p* = 0.16), GOBP_DOUBLE_STRAND_BREAK_REPAIR_VIA_SINGLE_STRAND_ANNEALING (*p* = 0.16), HP_INCREASED_SERUM_TESTOSTERONE_LEVEL (*p* = 0.16), GOMF_TESTOSTERONE_6_BETA_HYDROXYLASE_ACTIVITY (*p* = 0.24), HP_TRUNCAL_OBESITY (*p* = 0.24). A bubble plot of these selected pathways is shown in Figure 4.

Notably, many relevant gene pathways did not meet the threshold for significant differences between the two clusters. Examples of these included: HP_INSULIN_RESISTANCE, GOBP_REGULATION_OF_INSULIN_RECEPTOR_SIGNALING_PATHWAY, GOBP_TORC2_SIGNALING, PID_MTOR_4PATHWAY, PID_MYC_PATHWAY, and PID_PI3KCI_AKT_PATHWAY. 

**Figure 5:**
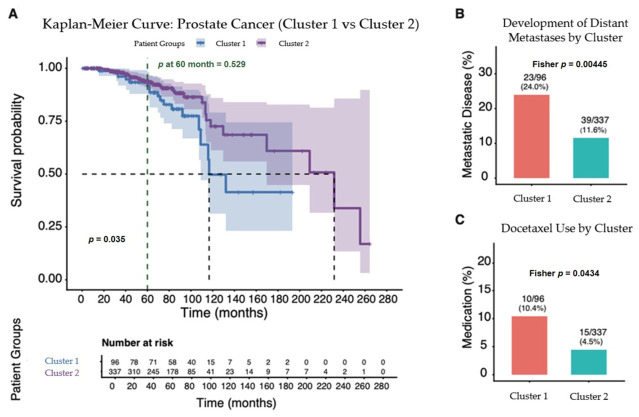
(**A**) Kaplan–Meier survival curve comparing the survival probabilities of the two patient clusters. Cluster 1 (blue line) has lower survival probability compared to cluster 2 (purple line). Median survival for cluster 1 is approximately 120 months, and 232 months in cluster 2. The *p*-value of 0.035 indicates a statistically significant difference in survival between the two groups. Also shown is a 60-month landmark with a *p*-value of 0.529 (not significant). This 60-month landmark was chosen as a reference point because it represents a time when a relatively large number of patients were still being considered in the analysis. (**B**) Box plot showing the percentage of patients in each cluster that developed distant metastases over time; 24.0% of patients in cluster 1 developed distant metastases compared to 11.6% in cluster 2 (*p* = 0.004). (**C**) Similar box plot showing docetaxel use in each cluster. Cluster 1 had 10.4% of patients go on to use docetaxel, and cluster 2 had 4.5% (*p* = 0.04).

We observed a median overall survival of 120 months and 232 months for cluster 1 and cluster 2, respectively (*p* < 0.05) (Figure 5A). Overall survival at 60-months showed no significant difference with a *p*-value of 0.529 (Figure 5A).This 60-month landmark was chosen as a reference point because it represents a time when a relatively large number of patients were still being considered in the analysis. A significant difference was found in the percentage of patients in each cluster that developed distant metastases over time, with 24.0% of those cluster 1 and 11.6% in cluster 2 (*p* = 0.004) (Figure 5B). Similarly, there was a significant difference in docetaxel use between the groups, with 10.4% of cluster 1 patients going on to use docetaxel and 4.5% of cluster 2 patients (*p* = 0.04) (Figure 5C).

The relative percentage of T3 and N1 tumors was significantly greater in cluster 1 compared to cluster 2 (*p* < 0.05). No significant difference was observed between any of the other clinical features assessed. Notably, there was no significant difference in age, race, hypertension, hypercholesterolemia, insulin-dependent diabetes, or BMI (Table 1).

**Figure 6:**
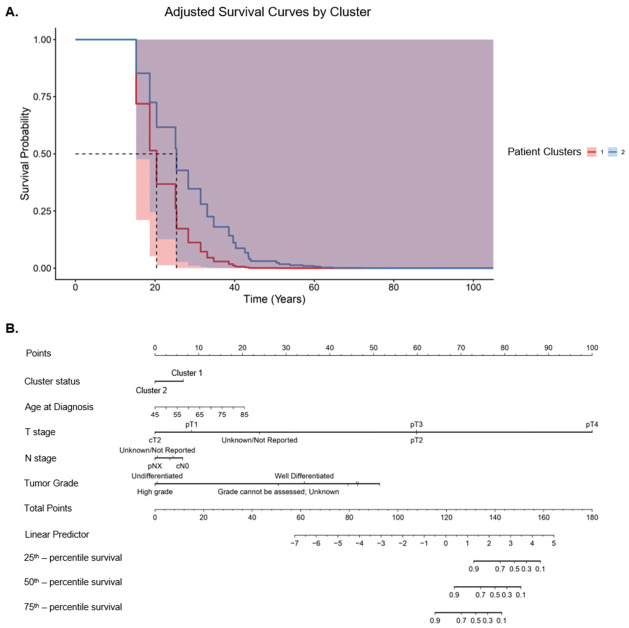
(**A**) KM curve between clusters 1 and 2 adjusted for potential confounders using Cox analysis. The potential confounders included: age at diagnosis, T stage, N stage, and tumor pathologic grade. Though a *p*-value could not be generated, the overlap between curves suggests no difference. (**B**) Nomogram showing the relative impact of each co-variate chosen, using Cox proportional hazards. T stage and pathologic grade had the greatest impact on survival. Cluster status corresponded to lower relative impact.

Figure 6A shows that the KM curve adjusted for confounders (age, T stage, N stage, and tumor pathologic grade) did not reveal differences in overall survival. Figure 6B goes on to show that the most significant co-variates associated with survival were tumor T stage and pathologic grade. Cluster status has an impact, but it is smaller than the aforementioned factors.

The results of the Cox analysis suggested that the survival difference between clusters was mediated by factors other than cluster status. T and N stages seemed to be the most likely explanation based on the fact that the T and N stages of tumors differed at baseline between patients in the two clusters. T and N stages also occupied a large portion of the nomogram in the Cox analysis. However, the directionality of the T and N relationship with cluster status remained unclear. For this reason, we conducted a mediation analysis.

The mediation analysis showed the following with respect to T stage and cluster status: cluster 2 status is associated with lower odds of having higher T stage (−1.43, *p* = 0.0029), cluster 2 status is associated with better survival independent of T stage with a HR of 0.53 (*p* = 0.039), and T stage does not significantly predict survival in this model (HR 1.39, *p* = 0.47). The total effect of cluster status on survival is −0.668 (*p* = 0.026), with a direct effect of −0.628 (*p* = 0.040) and an indirect effect of −0.040 (*p* = 0.41). The proportion mediated is −4.3% (*p* = 0.48).

**Figure 7:**
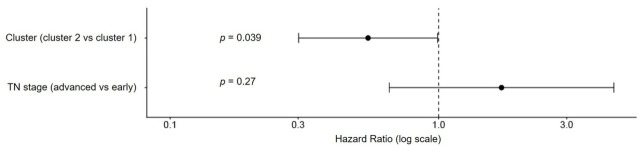
Forest plot demonstrating mediation analysis conducted between cluster status and TN stage. There is a significant association with cluster status and survival (*p* = 0.039), with cluster 2 status being associated with improved survival. TN stage is not significantly associated with survival (*p* = 0.27).

A similar mediation analysis was performed to assess the association with N stage and cluster status. It showed that cluster 2 status was not significantly associated with a lower odds of higher N stage (−0.459, *p* = 0.073). Cluster 2 was found to be associated with better survival once again, with a HR of 0.53 (*p* = 0.039). Higher N stage was not significantly associated with worse survival (HR = 1.36, *p* = 0.316). The total effect of cluster on survival was −0.658 (*p* = 0.029), with a direct effect of −0.629 (*p* = 0.039) and an indirect effect of −0.030 (*p* = 0.406). The proportion mediated was −3.2% (*p* = 0.47). Figure 7 shows a Forest plot demonstrating the effect of cluster status (*p* = 0.039) and TN stage on survival (*p* = 0.27).

## 4. Discussion

Our study identified two transcriptomic clusters in localized prostate tumors that demonstrated biologically plausible differences in insulin pathway genes. Cluster 1 embodied forms of insulin pathway dysregulation consistent with what had been previously described in the literature. In the unadjusted analysis, cluster 1 demonstrated reduced overall survival compared to cluster 2. This analysis also showed that cluster 1 had a greater proportion of patients who went on to need chemotherapy or develop distant metastases. Notably, the difference in overall survival between clusters was not observed after adjusting for confounders, implying that these confounders are what accounted for the survival difference rather than cluster status.

Cluster 1 demonstrated low levels of *PTEN* expression, consistent with *PTEN* mutational loss that has often been described as an early change in prostate cancers [34]. Additionally, cluster 1 shows high expression of *AKT* and *MTOR*, which are located downstream of *PI3K* [35]. The upregulation of *AKT* and *MTOR* is characteristic of PI3k-AKT-mTOR pathway dysfunction, with *MTOR* being a powerful oncogene [35]. *AKT* upregulation was the basis for the aforementioned clinical trials involving *AKT* inhibitors, CAPItello-281 and IPATential150, although these trials were in advanced disease, and this study is focused only on localized disease [29,30]. Low expression of *PIK3R1* was observed in cluster 1; *PIK3R1* loss has been previously cited as a frequent driver mutation following loss of *PTEN* [18]. High *IGFBP2* expression was also seen in cluster 1, which has been previously found to track with *PTEN* loss, so much so that it has been proposed as a surrogate for *PTEN* activity [36].

Additional gene expression changes were identified that were consistent with previous studies, though the literature has been more limited in scope. Elevated expression of *IRS2* was observed in cluster 1. IRS2 acts directly downstream of the insulin receptor and proximally to PTEN and the PI3K-AKT-mTOR pathway [37]. IRS2 has been associated with enhanced cell growth and migration in cell culture models [37]. A specific mutation in *IRS1* was associated with an increased risk for developing prostate cancer and more advanced disease [38].

One notable difference was that *AKT1* was upregulated in cluster 1, while *AKT3* was downregulated in the same cluster. This finding supports differential roles for different *AKT* isoforms, which has been suggested by prior research [39]. These differences in *AKT* isoforms may have relevance for future drug development, as the CAPItello-281 and IPATential150 utilized a pan-*AKT* inhibitor targeting all three *AKT* isoforms (*AKT* 1–3) [29,30].

Another interesting finding is the predominance of expression changes in the lipid metabolism genes in cluster 1. The top contributor to cluster 1 is *FASN* expression, which stands out at an expression of over 3.0 × 10^7^, while every other contributing gene is less than 1.0 × 10^7^. Prostate cancers are known to utilize lipid metabolism early in the course of the disease and to an unusual extent compared with other cancers [40]. In this regard, primary prostate tumors are not easily detectable by 8F-FDG-PET because they do not undergo aerobic glycolysis at a rate significantly greater than non-malignant cells [40]. However, prostate cancers are readily detectable by PET using radioactive lipid metabolism substrates rather than glucose, such as acetate or choline [40,41]. Androgen signaling has crosstalk with lipid metabolism gene expression, and it has been shown that as patients develop castrate resistance, lipid metabolism genes are upregulated [42]. The expression of lipid genes, including *FASN*, has been associated with migration and invasion in prostate cancer models and with shorter overall survival in *PTEN*-deficient prostate cancers [43,44]. The proposed mechanism of *FASN*’s role in cancer progression is multi-factorial, related to its ability to produce membrane lipids necessary for rapid cell proliferation, inhibition of apoptosis, and facilitation of *B*-catenin signaling (which has a crucial role in EMT) [45,46]. Notably, the third and thirteenth top contributors to cluster 1 are also lipid metabolism genes: *ACACA* and *SREBF1*. *ACACA* catalyzes the rate-limiting step in fatty acid synthesis, and *SREBF1* is a key transcription factor regulating a host of lipid metabolism genes [47].

GSEA analysis, typified in the bubble plot (Figure 4), demonstrated several unexpected findings. Cluster 1 demonstrated alterations in WNT secretion, which has been implicated in more aggressive disease, androgen resistance, and neuro-endocrine phenotypes [48,49]. Cluster 1 was also associated with transcriptome changes characteristic of *TMPRSS2*-*ERG* fusion. This is a common genetic alteration in prostate cancer associated with poor survival and more aggressive disease [50]. It commonly co-occurs with PTEN loss, which is consistent with our gene expression findings described earlier [24]. Perhaps less unexpected were alterations in a number of lipid metabolism pathways that also occurred in cluster 1. These pathway changes are consistent with the alterations in *ACACA*, *SREBF1*, and *FASN* described above.

The GSEA analysis showed a number of pathway changes associated with cluster 2. Many of these involved DNA damage repair and varied from DNA damage sensing to mismatch repair to repair of double-stranded breaks. DNA damage repair mutations are common in advanced prostate cancer, particularly in genes involved in homologous recombination repair [51]. These mutations predispose to more aggressive disease, though they can sometimes represent the targets of advanced therapies such as olaparib for *BRCA1/2* [52]. Cluster 2 was also enriched for transcriptomic changes characteristic of elevated testosterone. High levels of androgens like testosterone promote tumor growth in prostate cancer [53]. Interestingly, there is crosstalk between androgen signaling and DNA-damage repair, such that elevated androgen levels work to promote DNA repair [54].

A notable negative finding in the GSEA analysis was that there was no significant difference in the insulin resistance pathway between the two groups. Though cluster 1 certainly seemed to have at least some gene expression changes that are typically associated with insulin sensitivity (such as *PTEN* loss), the more widespread differences that might be expected were not seen. It may be that the pattern of dysregulation in these tumors is such that, while the insulin receptor may be more active, the subsequent downstream pathways are disrupted in a way that is different from what would be expected in an insulin-sensitive, though non-malignant, cell. It may also be that changes in insulin resistance were partially confounded by differences in the prevalence of non-insulin-dependent type two diabetes between the two populations. Only data on insulin-dependent diabetics was available for inclusion in our analysis.

Overall survival was reduced in cluster 1 compared to cluster 2 in the unadjusted analysis. An important caveat is that there was no difference in overall survival at our 60-month landmark. This landmark was chosen because it represents a point at which the number of patients being compared remains substantial. At 60 months, 58 patients in cluster 1 were compared with 178 in cluster 2. Later on in the KM curve, the number of patients being compared between the groups drops off. By 180 months, two patients in cluster 1 are being compared to seven in cluster 2. While a significant difference in overall survival was found on the whole, it should be noted that we cannot rule out random differences from the low number of patients being compared late in the KM curve as having played a role. It is also worth noting that this KM curve did not take into account other variables, such as tumor stage, that were found to differ in the descriptive table.

The increased percentages of patients in cluster 1 that demonstrated greater use of docetaxel and greater development of distant metastases are supportive of more aggressive disease present in cluster 1. Like the KM curve findings, however, these did not account for other variables that differed between the patients.

The patient characteristics demonstrated in Table 1 showed that patients in the two clusters were similar and different in important ways. The two groups did not differ statistically in age, race, hypertension, hyperlipidemia, insulin-dependent diabetes, or BMI. However, they did differ significantly in tumor stage at diagnosis. Cluster 1 consistently had more advanced disease at diagnosis, as demonstrated by a greater percentage of both T4 and N1 tumors.

The Cox analysis in Figure 6 showed that, in fact, the difference in overall survival between clusters 1 and 2 was erased when the following confounders were controlled for: age at diagnosis, T stage, N stage, and tumor pathologic grade. The associated nomogram based on this analysis shows that T stage and pathologic grade represent the highest impact co-variates. On the whole, this implies that the differences in survival between clusters can be explained by other differences between patients. As the difference in survival initially seen with the cluster analysis disappeared after controlling for co-variates, cluster status is not an independent prognostic factor. The RNA changes that we identified may simply be representative of other differences between tumors, such as pathologic grade, rather than reflecting other underlying biology.

The clinical differences between T and N stage between the clusters at baseline, as well as the large representation of T and N stage on the Cox nomogram, caused us to hypothesize that differences in T and N stage may explain the difference in overall survival observed between the two clusters. To test this hypothesis, we conducted a mediation analysis. This analysis showed that there was a significant association between cluster status and overall survival. However, T and N stages were not significantly associated with a difference in overall survival. There was no significant mediation effect through either T or N stage. This finding implies that confounders other than T or N stage were likely responsible for the overall survival difference between clusters. It seems most likely that these confounders would be the other factors assessed in the Cox analysis: pathologic grade and age at diagnosis. Of course, other unaccounted confounders could also play a role.

Though our research provided a number of insights, there were certain limitations. The size of the study was sufficient for the analysis of overall survival but lacked the data to provide a robust analysis of other relevant clinical outcomes. The focus on overall survival leaves significant room for known and unknown confounding biases. The use of RNA expression data alone is another limitation and makes our findings hypothesis-generating rather than confirmatory. Further validation is warranted to confirm these findings with the use of whole-exome sequencing to provide additional DNA-level context for the differences in gene expression observed. For example, a drop in *PTEN* expression may have been aligned with a concurrent increase in *PTEN* genomic loss between clusters. This would help strengthen connections between the expression changes and the existing literature, which more heavily cites DNA alterations. Other types of validation, such as use of an independent transcriptomic cohort or protein-level confirmation of key markers like PTEN or mTOR, could also provide further support. Finally, while the molecular programs identified have been implicated in disease progression in other contexts, this analysis was limited to localized disease at diagnosis and did not evaluate subsequent metastatic outcomes due to a lack of available data in the dataset.

This research supports future studies that are optimally designed to assess potential correlation of insulin pathway changes at the molecular level and clinical outcomes. Future studies that provide additional modes of concurrent molecular analysis are also warranted (RNA sequencing, whole-exome sequencing, etc.).

## 5. Conclusions

Overall, this study identified a subset of patients with localized prostate cancer who enriched for insulin pathway alterations. Although on unadjusted analysis the cluster with molecular changes suggestive of insulin pathway dysregulation demonstrated reduced overall survival, this survival difference disappeared when controlling for confounders. Our mediation analysis showed that tumor stage was, in fact, not the most likely cause of the survival difference. It is likely that other confounders, such as age at diagnosis or pathologic grade, played a role. Our findings are consistent with prior studies that suggest early insulin pathway dysregulation may contribute to risk of metastasis and poor outcomes in prostate cancer. The gene expression changes we identified align with prior molecular studies while suggesting additional relevant changes not previously mentioned. However, with transcriptomic data alone, these findings are hypothesis-generating. This research supports additional confirmatory studies to further delineate the clinical implications of molecular dysregulation of insulin pathways in localized prostate cancer.

## Figures and Tables

**Table 1:** Descriptive table demonstrating various patient characteristics in cluster 1 and cluster 2. A *p*-value is listed, indicating statistical significance if <0.05. Significant differences were identified between tumor T stage and N stage. “C” indicates clinical staging and “*p*” indicates pathologic staging. “NOS” indicates Not Otherwise Specified.

	Cluster 1 (*n* = 96)	Cluster 2 (*n *= 337)	*p*-Value
Age at clinical record, median (IQR, min, max)	68.018 (10.1, 51.3, 84.8)	66.248 (10.42, 47.72, 85.61)	0.075
T stage, n (%)			0.005
cT2	0 (0%)	2 (0.005%)	
cT3	1 (0.01%)	0 (0%)	
pT0	1 (0.01%)	0 (0%)	
pT1	0 (0%)	2 (0.005%)	
pT2	5 (0.05%)	58 (17.2%)	
pT3	88 (91.6%)	271 (80.4%)	
pT4	1 (0.01%)	2 (0.005%)	
Unknown/Not Reported	0 (0%)	2 (0.005%)	
N stage:			0.009
cN0	1 (0.01%)	10 (0.02%)	
pN0	56 (58.3%)	240 (71.2%)	
pN1	30 (31.2%)	77 (22.8%)	
pNX	9 (0.09%)	9 (0.02%)	
Unknown/Not Reported	0 (0%)	1 (0.003%)	
Race, n (%)			0.825
Asian Indian	0 (0%)	1 (0.003%)	
Asian Indian, NOS or Pakistani, NOS	0 (0%)	1 (0.003%)	
Black or African American	6 (0.06%)	28 (0.08%)	
Chinese	0 (0%)	1 (0.003%)	
Filipino	0 (0%)	1 (0.003%)	
Korean	0 (0%)	1 (0.003%)	
Other Asian, including Asian, NOS and Oriental NOS	1 (0.01%)	4 (0.01%)	
Some other race	1 (0.01%)	3 (0.009%)	
Unknown by patient	1 (0.01%)	2 (0.005%)	
Vietnamese	1 (0.01%)	0 (0%)	
White	86 (89.5%)	295 (87.5%)	
Hypertension, n (%)			0.245
Yes	50 (52.0%)	198 (58.7%)	
No	46 (47.9%)	139 (41.2%)	
Hypercholesterolemia, n (%)			0.801
Yes	16 (16.6%)	53 (15.7%)	
No	80 (83.3%)	280 (83.0%)	
Unknown/Not Applicable	0 (0.0%)	4 (0.01%)	
IDDM, n (%)			0.619
Yes	4 (0.04%)	20 (0.06%)	
No	92 (95.8%)	317 (94.0%)	
BMI > 30, median (IQR, min, max)	34.000 (3.54, 30.2, 39.58)	33.275 (4.27, 30.1, 39.9)	0.581

## Data Availability

Restrictions apply to the availability of these data. Data were obtained from ORIEN and are available from https://www.oriencancer.org with the permission of the organization. URL accessed on 8 May 2025.

## Supplementary Materials

The following supporting information can be downloaded at: https://www.mdpi.com/article/10.3390/cancers18101636/s1, Figure S1. (A) Silhouette plot showing the distribution of clusters for k = 2. Cluster 1 is shown in red on the left, and cluster 2 is shown in blue on the right. (B) Silhouette plot showing the distribution for clusters with k = 3. Clusters 1, 2, and 3 are shown in red, green, and blue, respectively. Figure S2. Scatter plot showing the distribution of patients across clusters 1 and 2. In this analysis, 23 patients (shown in red) were identified with intermediate status between clusters 1 and 2. These patients were deemed outliers and filtered from the analysis. Table S1. Table showing full set of C2 pathway changes below the FDR threshold of 0.25. The pathway ID, name, log fold change, *p*-value, and FDR are shown. Table S2. Table showing full set of C5 pathway changes below the FDR threshold of 0.25. The pathway ID, name, log fold change, *p*-value, and FDR are shown.

## Abbreviations

The following abbreviations are used in this manuscript:

ORIEN	Oncology Research Information Exchange Network
NMF	Non-negative Matrix Factorization
GSEA	Gene Set Enrichment Analysis
AR	Androgen Receptor
EMT	Epithelial-to-Mesenchymal Transition
KM	Kaplan–Meier
PFS	Progression Free Survival
rPFS	Radiographic Progression Free Survival
mCRPC	Metastatic Castrate Resistant Prostate Cancer
mHSPC	Metastatic Hormone Sensitive Prostate Cancer
TCC	Total Cancer Care
GURIG	Genitourinary Research Interest Group
WES	Whole Exome Sequencing
BMI	Body Mass Index
OS	Overall Survival
NOS	Not Otherwise Specified
IDDM	Insulin-Dependent Diabetes Mellitus
