# Supplementary material for: Insulin Pathway Changes in Localized Prostate Cancer: A Multi-Institutional Analysis
Source: Cancers (Basel). 2026 May 19;18(10):1636. doi: 10.3390/cancers18101636 (PMC13205060; doi:10.3390/cancers18101636)
Supplement: Supplementary file 1 [file cancers-18-01636-s001.zip › cancers-4256307-supplementary.pdf]

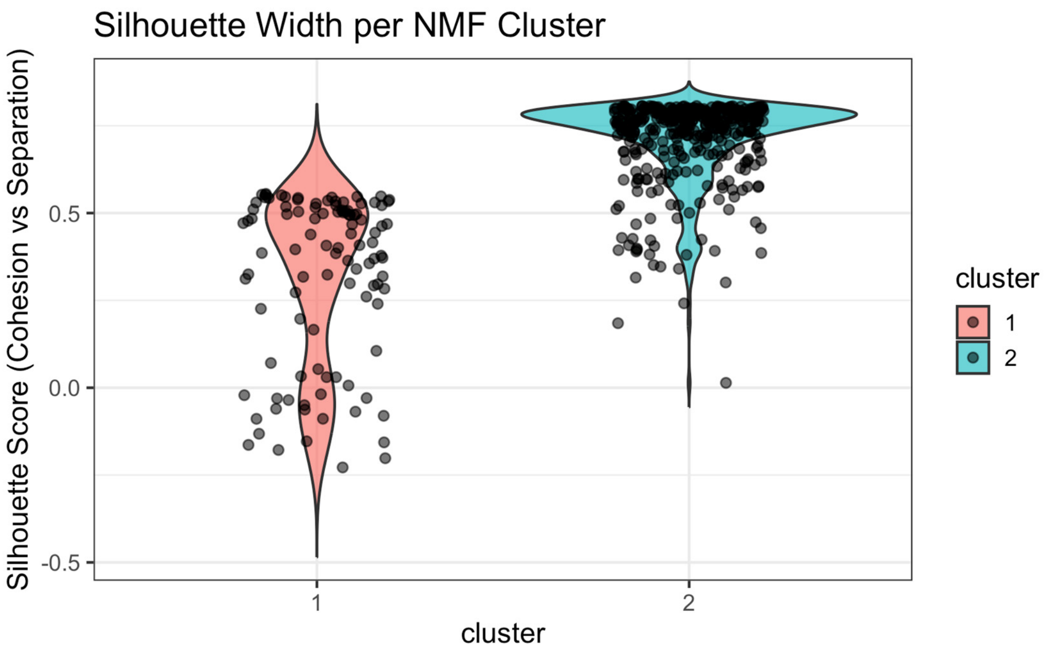

(a)

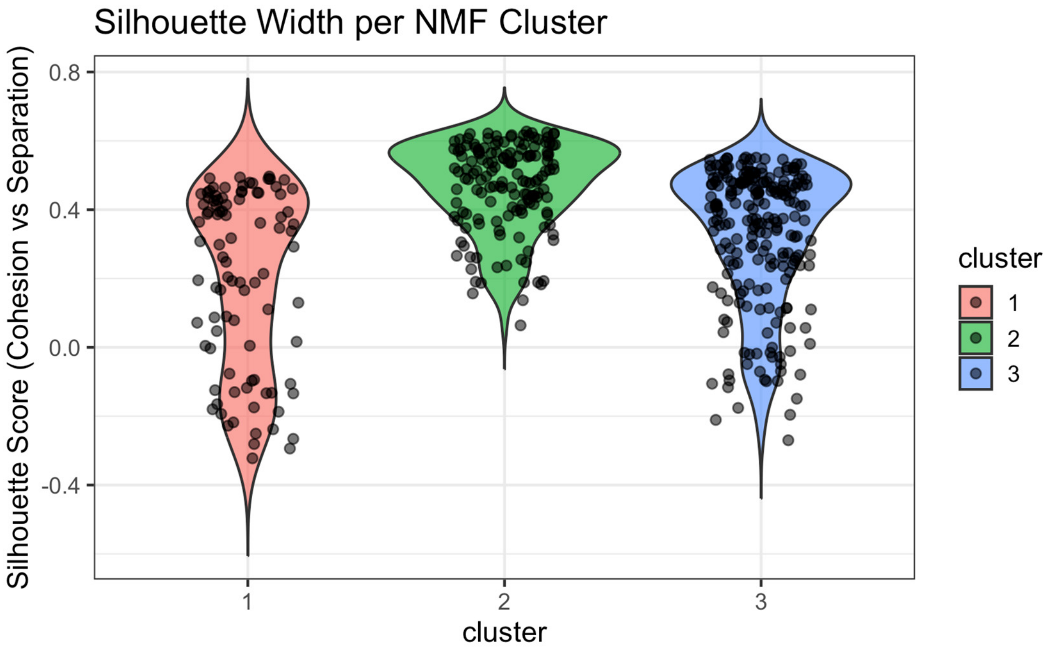

(b)

Figure S1. A) Silhouette plot showing the distribution of clusters for k = 2. Cluster 1 is shown in red on the left, and cluster 2 is shown in blue on the right. (B) Silhouette plot showing the distribution for clusters with k = 3. Clusters 1, 2, and 3 are shown in red, green, and blue, respectively.

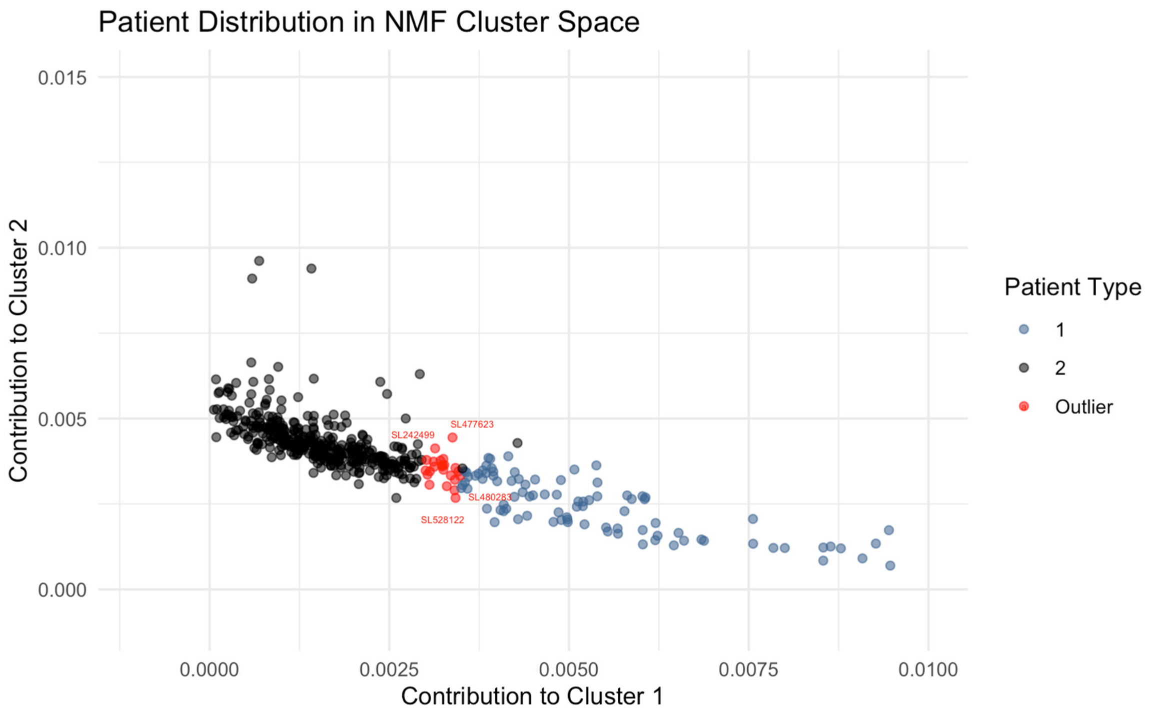

Figure S2. Scatter plot showing the distribution of patients across clusters 1 and 2. In this analysis, 23 patients (shown in red) were identified with intermediate status between clusters 1 and 2. These patients were deemed outliers and filtered from the analysis.

Table S1. Table showing full set of C2 pathway changes below the FDR threshold of 0.25. The pathway ID, name, log fold change, p-value, and FDR are shown.

ID	Pathway Name	Log fold change	p-value	FDR
2957	LUND_SILENCED_BY_METHYLATION	-0.152	0.000162	0.192
5898	SETLUR_PROSTATE_CANCER_TMPRSS2_ERG_FUSION_UP	-0.152	0.000157	0.192
678	CAFFAREL_RESPONSE_TO_THC_8HR_3_UP	-0.119	0.000103	0.192
5165	REACTOME_RHO_GTPASES_ACTIVATE_KTN1	-0.094	0.000228	0.192
3845	RAMPON_ENRICHED_LEARNING_ENVIRONMENT_EARLY_UP	-0.085	0.000227	0.192
6350	WANG_RESPONSE_TO_GSK3_INHIBITOR_SB216763_UP	0.077	0.000215	0.192
7556	ZWANG_DOWN_BY_2ND_EGF_PULSE	0.107	0.000076	0.192
5999	SPIRA_SMOKERS_LUNG_CANCER_DN	0.107	0.000054	0.192
6347	WANG_RESPONSE_TO_FORSKOLIN_DN	0.118	0.000111	0.192
1565	HUTTMANN_B_CLL_POOR_SURVIVAL_DN	0.091	0.000321	0.243

Table S2. Table showing full set of C5 pathway changes below the FDR threshold of 0.25. The pathway ID, name, log fold change, p-value, and FDR are shown.

ID	Pathway Name	Log fold change	p-value	FDR
11544	HP_ABNORMALITY_OF_THE_LEYDIG_CELLS	0.128	0.000007	0.073
15493	HP_SCHWANNOMA	0.105	0.000009	0.073
171	GOBP_AMYLOID_BETA_CLEARANCE_BY_TRANSCYTOSIS	-0.152	0.000026	0.126
2270	GOBP_LONG_CHAIN_FATTY_ACID_IMPORT_INTO_CELL	-0.111	0.000038	0.126
7562	GOBP_WNT_PROTEIN_SECRETION	-0.171	0.000044	0.126
5844	GOBP_REGULATION_OF_LONG_CHAIN_FATTY_ACID_IMPORT_INTO_CELL	-0.169	0.000062	0.126
9091	GOMF_DNA_DAMAGE_SENSOR_ACTIVITY	0.136	0.000074	0.126
12938	HP_DYSMETRIC_SACCADES	-0.072	0.000075	0.126
13156	HP_EUTHYROID_GOITER	0.231	0.000076	0.126
6477	GOBP_REGULATION_OF_WNT_PROTEIN_SECRETION	-0.177	0.000078	0.126
8807	GOMF_ATP_DEPENDENT_DNA_DAMAGE_SENSOR_ACTIVITY	0.179	0.000089	0.131
15032	HP_POSTAXIAL_FOOT_POLYDACTYLY	0.076	0.000135	0.156
14767	HP_OVARIAN_NEOPLASM	0.068	0.000144	0.156
13459	HP_GONADAL_NEOPLASM	0.055	0.000161	0.156
2574	GOBP_MISMATCH_REPAIR	0.095	0.000161	0.156
1236	GOBP_DOUBLE_STRAND_BREAK_REPAIR_VIA_SINGLE_STRAND_ANNEALING	0.116	0.000162	0.156
10855	HP_ABNORMAL_FIFTH_TOE_MORPHOLOGY	0.074	0.000166	0.156
13014	HP_ELEVATED_CIRCULATING_PARATHYROID_HORMONE_LEVEL	0.092	0.000176	0.156
14764	HP_OVARIAN_CARCINOMA	0.139	0.000196	0.156
15545	HP_SEVERE_INTRAUTERINE_GROWTH_RETARDATION	0.133	0.000208	0.156
5843	GOBP_REGULATION_OF_LONG_CHAIN_FATTY_ACID_IMPORT_ACROSS_PLASMA_MEMBRANE	-0.174	0.000209	0.156
12087	HP_ATYPICAL_NEVI_IN_NON_SUN_EXPOSED_AREAS	0.178	0.000226	0.156
13921	HP_INCREASED_SERUM_TESTOSTERONE_LEVEL	0.099	0.000232	0.156
15961	HP_TONGUE_NODULES	0.084	0.000239	0.156
15021	HP_POPLITEAL_PTERYGIUM	0.168	0.000242	0.156
1205	GOBP_DNA_STRAND_INVASION	0.171	0.000260	0.156
8293	GOCC_PERINUCLEOLAR_COMPARTMENT	-0.144	0.000263	0.156
2236	GOBP_LIPID_IMPORT_INTO_CELL	-0.089	0.000269	0.156
1464	GOBP_ESTABLISHMENT_OF_SISTER_CHROMATID_COHESION	0.123	0.000306	0.171
5612	GOBP_REGULATION_OF_ENDOSOME_TO_PLASMA_MEMBRANE_PROTEIN_TRANSPORT	0.124	0.000368	0.186
8870	GOMF_CADHERIN_BINDING	-0.056	0.000368	0.186
1247	GOBP_EARLY_ENDOSOME_TO_RECYCLING_ENDOSOME_TRANSPORT	-0.096	0.000370	0.186
918	GOBP_COBALAMIN_METABOLIC_PROCESS	0.102	0.000378	0.186
11011	HP_ABNORMAL_MEGAKARYOCYTE_MORPHOLOGY	0.062	0.000431	0.195
4172	GOBP_POSITIVE_REGULATION_OF_CENTRIOLE_ELONGATION	0.117	0.000437	0.195
7228	GOBP_TELOMERIC_D_LOOP_DISASSEMBLY	0.097	0.000448	0.195
1600	GOBP_FORMATION_OF_EXTRACHROMOSOMAL_CIRCULAR_DNA	0.093	0.000453	0.195
14326	HP_MEGACOLON	0.052	0.000457	0.195
5042	GOBP_PROTEIN_LOCALIZATION_TO_CILIARY_TRANSITION_ZONE	0.110	0.000485	0.202
3878	GOBP_PEPTIDYL_THREONINE_DEPHOSPHORYLATION	-0.094	0.000544	0.220
12897	HP_DORSOCERVICAL_FAT_PAD	0.078	0.000660	0.240
14198	HP_LOBAR_HOLOPROSENCEPHALY	0.069	0.000670	0.240
9192	GOMF_FOUR_WAY_JUNCTION_DNA_BINDING	0.095	0.000710	0.240
13077	HP_ENLARGED_SYLVIAN_CISTERN	0.105	0.000716	0.240

16012	HP_TYPE_2_MUSCLE_FIBER_PREDOMINANCE	0.080	0.000719	0.240
3938	GOBP_PHOSPHATIDYLSERINE_ACYL_CHAIN_REMODELING	-0.095	0.000729	0.240
4410	GOBP_POSITIVE_REGULATION_OF_ISOTYPE_SWITCHING_TO_IGG_ISOTYPES	0.100	0.000785	0.240
5060	GOBP_PROTEIN_LOCALIZATION_TO_NON_MOTILE_CILIUM	0.126	0.000807	0.240
14992	HP_POIKILODERMA	0.072	0.000818	0.240
1202	GOBP_DNA_REPLICATION_SYNTHESIS_OF_PRIMER	0.129	0.000820	0.240
8853	GOMF_BITTER_TASTE_RECEPTOR_ACTIVITY	0.216	0.000835	0.240
14836	HP_PARIETAL_BOSSING	0.120	0.000839	0.240
14459	HP_MULTIPLE_CAFE_AU_LAIT_SPOTS	0.067	0.000843	0.240
13601	HP_HYPERCHLOREMIC_ACIDOSIS	0.108	0.000855	0.240
14468	HP_MULTIPLE_LENTIGINES	0.106	0.000867	0.240
12482	HP_COLDNESS	0.173	0.000893	0.240
8656	GOMF_5_3_DNA_HELICASE_ACTIVITY	0.143	0.000894	0.240
5487	GOBP_REGULATION_OF_CELLULAR_PH_REDUCTION	0.100	0.000903	0.240
12274	HP_CAFE_AU_LAIT_SPOT	0.044	0.000909	0.240
12566	HP_CORNEAL_GUTTATA	0.194	0.000913	0.240
3361	GOBP_NEGATIVE_REGULATION_OF_PROTEIN_TARGETING_TO_MEMBRANE	0.127	0.000924	0.240
12119	HP_BASAL_CELL_CARCINOMA	0.058	0.000938	0.240
14332	HP_MEGALOBLASTIC_ANEMIA	0.072	0.000978	0.240
14218	HP_LONG_THUMB	0.136	0.000991	0.240
7856	GOCC_DENSE_CORE_GRANULE_LUMEN	0.178	0.001019	0.240
15815	HP_SUDDEN_UNEXPECTED_DEATH_IN_EPILEPSY	0.126	0.001034	0.240
10316	GOMF_TESTOSTERONE_6_BETA_HYDROXYLASE_ACTIVITY	0.205	0.001038	0.240
5498	GOBP_REGULATION_OF_CENTRIOLE_ELONGATION	0.092	0.001043	0.240
14735	HP_OROFACIAL_DYSKINESIA	0.072	0.001090	0.240
7288	GOBP_TOOTH_ERUPTION	0.157	0.001090	0.240
13650	HP_HYPERPHOSPHATEMIA	0.116	0.001100	0.240
2269	GOBP_LONG_CHAIN_FATTY_ACID_IMPORT_ACROSS_PLASMA_MEMBRANE	-0.136	0.001103	0.240
6034	GOBP_REGULATION_OF_NODAL_SIGNALING_PATHWAY	-0.163	0.001112	0.240
14032	HP_IRREGULAR_MENSTRUATION	0.064	0.001118	0.240
11127	HP_ABNORMAL_PELVIS_BONE_MORPHOLOGY	0.081	0.001136	0.240
4409	GOBP_POSITIVE_REGULATION_OF_ISOTYPE_SWITCHING	0.056	0.001144	0.240
11683	HP_ABSENT_PUBERTAL_GROWTH_SPURT	0.217	0.001150	0.240
4750	GOBP_POSITIVE_REGULATION_OF_STRESS_GRANULE_ASSEMBLY	-0.089	0.001156	0.240
16002	HP_TRUNCAL_OBESITY	0.066	0.001190	0.244
